# Impact of Storage Temperature on Pollen Viability and Germinability of Four Serbian Autochthon Apple Cultivars

**DOI:** 10.3389/fpls.2021.709231

**Published:** 2021-07-29

**Authors:** Dušica Ćalić, Jelena Milojević, Maja Belić, Rade Miletić, Snežana Zdravković-Korać

**Affiliations:** ^1^Department of Plant Physiology, Institute for Biological Research “Siniša Stanković, ” National Institute of Republic of Serbia, University of Belgrade, Belgrade, Serbia; ^2^Fruit Research Institute-Čačak, Čačak, Serbia

**Keywords:** apple, cultivars, pollen germination, pollen storage, viability

## Abstract

Globalization has drastically reduced the number of autochthon apple cultivars in the Serbian market and most of them have nearly disappeared; however, some of these cultivars, such as Petrovača, Budimka, Kolačara Pozna, and Kožara, have extraordinary quality, good pomological characteristics, and pest and disease resistance. The present study was conducted to develop a protocol for the storage of pollen for further use in the conservation and breeding of these cultivars. Viability and germination of the mature pollen were tested *in vitro*, at four storage temperatures (20, 4, −20, and −80°C), right after harvest or 1, 2, 3, 4, 5, and 6 months after storage. Differences in fresh pollen viability and germination between cultivars were statistically significant and ranged from 60 to 88% and 59 to 98%, respectively. Fresh pollen of cv. Budimka showed the highest viability and germination in comparison with other cultivars, especially cv. Kožara. Pollen viability and germination decreased over the storage period, and it was the lowest after 6 months of storage at room temperature in all tested cultivars. Storage at 4°C prolonged the pollen viability and germinability of 1–5 fold, depending on the cultivar and treatment duration; however, the pollen longevity of all cultivars was significantly extended when stored at −20 or −80°C. After 6 months, pollen of cv. Budimka stored at −20 and −80°C showed 14–15 fold higher germination rates in relation to pollen storage at room temperature for the same period. The results of the present study suggest that the pollen of these apple cultivars could be efficiently maintained at −20°C and could be further used for breeding purposes, e.g., for crossings between cultivars that flower at different times of the year.

## Introduction

Apple (*Malus* × *domestica* Borkh.) is one of the most important and widely cultivated fruit species around the world with over 7,500 cultivars (Elzebroek and Wind, 2008). China is the largest apple producer, while the USA is the second (FAO, 2016). It is the most produced fruit in temperate climate zones, but currently, its production is expanding into subtropical and tropical zones (Brown, 2012).

Globalization and standardization in the Serbian apple market have drastically reduced the numbers of autochthon cultivars and most of them have nearly disappeared; however, some of these cultivars, such as Petrovača, Budimka, Kolačara Pozna, and Kožara, have extraordinary quality, good pomological characteristics, and pest and disease resistance (Mratinić, 2005; Mratinić and Fotirić Akšić, 2011, 2012).

These autochthon apple cultivars were chosen because of the following features: cv. Budimka is resistant to spring frosts, cv. Petrovača gives fruits at the beginning of summer, cv. Kolačara has the tastiest fruits for cakes, and cv. Kozara has fruits suitable for long-term storage (Mratinić, 2005).

Apple is a self-incompatible and insect-pollinated species. Pollination is the key event for apple fruits production, and fertilization normally occurs among the cultivars. The production of pollen grains with high viability, suitable for storage and transport, is of great importance in selective breeding programs (Chagas et al., 2010). It advances the fertilization process and permits crossings between genotypes that flower at different times (Machado et al., 2014). Efficient pollen storage and high viability are also important for biodiversity, biotechnology, conservation, and other studies.

Several reports have shown that pollen stored at low temperatures was effective for long-term preservation, such as pollen of almond (Martínez-Gómez et al., 2002), mango (Dutta et al., 2013), hazel (Novara et al., 2017), and date palm (Maryam et al., 2017). Pollen stored at −20 or −196°C has extended longevity for at least a year (Bhat et al., 2012). The storage conditions, including temperature, have an immense effect on pollen viability (Deng and Harbaugh, 2004). Pollen age, physiological state of a flower as well as pollen moisture content affect pollen viability during storage. There is little data available in the literature on the storage of apple pollen at low temperatures, except for a few other apple cultivars (Imani et al., 2011).

The *in vitro* germination of pollen grains is the most commonly used viability assay in genetic improvement programs (Imani et al., 2011; Soares et al., 2013; Machado et al., 2014); however, each species requires a specific protocol and culture medium to obtain effective germination *in vitro* (Zambon et al., 2014). The viability of pollen grains could be also determined by indirect methods based on cytological observations following the staining of pollen grains with vital fluorescent dyes (Ćalić et al., 2013; Impe et al., 2020).

Currently, no report is available on the cold pollen storage of any Serbian autochthon apple cultivar. Therefore, the objective of this study was to investigate appropriate temperature conditions for long-term pollen storage of four selected Serbian apple cultivars for possible use in the breeding of these cultivars.

## Materials and Methods

### Plant Materials

Mature pollen of four Serbian autochthon apple cultivars (Petrovača, genotypes VI 49–51; Budimka, genotypes IV 1–3; Kolačara Pozna, genotypes VIII 10–12; and Kožara, genotypes IX 16–18) was harvested in spring 2016. The selected apple belongs to the fruit collection of Fruit Research Institute, Čačak, Serbia. The pollen was collected in the sterile Falcon tubes in the noon of the sunny days to collect samples with low humidity.

### Storage

To evaluate the best conditions for pollen storage and extended longevity, different storage periods (0, 1, 2, 3, 4, 5, and 6 months) and temperatures (20, 4, −20, and −80°C) were tested. Pollen was transferred from Falcon tubes to Petri dishes and dehydrated over silica gel at room temperature. Samples from Petri dishes were divided into several Eppendorf tubes to reduce the freeze-thaw stress. Eppendorf tubes were sealed and stored at the above-mentioned temperature treatments. After thawing, stored pollen samples were incubated at room temperature for 24 h.

### Fluorescein Diacetate Viability Test

Fluorescein diacetate (FDA) test, as a rapid test, was used to determine pollen viability (Heslop-Harrison and Heslop-Harrison, 1970). FDA (2 mg L^−1^), dissolved in acetone, was diluted by 0.5 M sucrose solution (1:1). Pollen grains were stained with 1–2 drops of FDA for 1 h, and then preparations were observed under the fluorescent Axiovert microscope (Zeiss, Jena, Germany) with fluorescence filters. Pollen grains showing bright fluorescence were taken as viable and scored.

### *In vitro* Pollen Germination

*In vitro* pollen germination was performed by using the hanging drop technique (Deng and Harbaugh, 2004; Ćalić et al., 2013) with some modifications as described below. A liquid medium containing 1.2 M sucrose, 0.3 g L^−1^ calcium nitrate [Ca(NO_3_)_2_], 0.10 g L^−1^ boric acid (H_3_BO_3_), 0.1 g L^−1^ potassium nitrate (KNO_3_), 0.2 g L^−1^ magnesium sulfate (MgSO_4_·7H_2_O), and 15 %, w/v polyethylene glycol (PEG) was used. A germination test was recorded after a 24 h-incubation period. Pollen was adjudged as having germinated when the length of the pollen tube was equal to or exceeded the pollen diameter. Pollen grains were counted under a light microscope (Leica DMRB, Wetzlar, Germany) at ×20 magnification. Germination of pollen was tested after harvest (0 times), and at 1, 2, 3, 4, 5, and 6 months after storage.

### Pollen Nucleus Status

Pollen nuclei number was determined by 4′, 6-diamidino-2-phenylindole (DAPI) (Coleman and Goffm, 1985). The content of anthers was removed by squeezing and was stained with 1–2 drops of DAPI (1 μg mL^−1^) solution prepared using distilled water. DAPI-treated microspores were examined under the Zeiss Axiovert fluorescent microscope equipped with a camera.

### Statistical Analysis

The measurements of the pollen germination and viability were taken on 300 randomly chosen pollen grains (in three repetitions, each with 100 pollen grains) per treatment. To analyze the main effects and interaction effects, the data were subjected to analysis of variance (three-way ANOVA), and the means were separated using an LSD test at *p* ≤ 0.05.

## Results

### Fresh Pollen Characteristics

**Figure 1:**
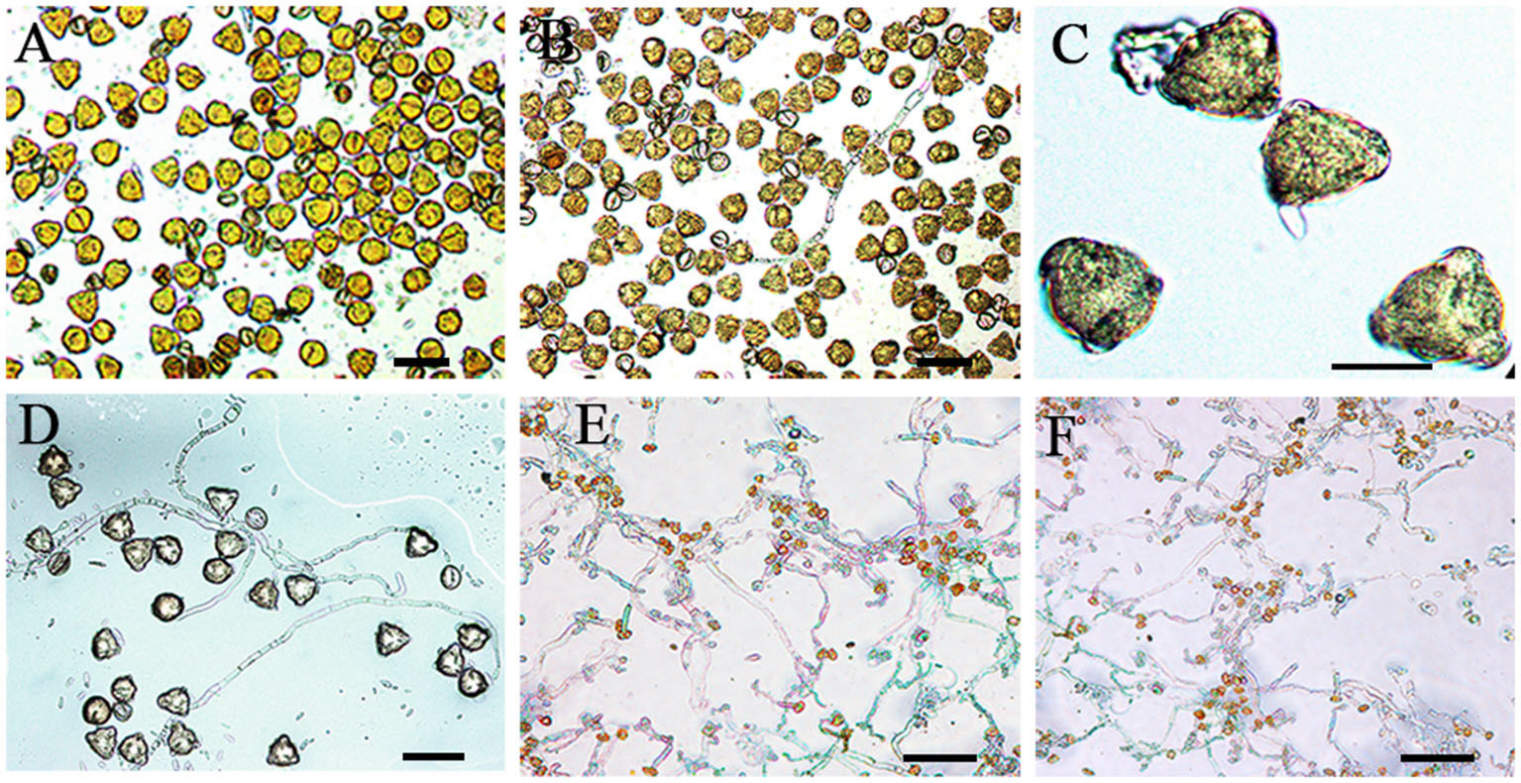
Pollen germination after storage treatment. **(A)** Pollen germination of cv. Kožara at + 20°C (bar = 50 μm), at +4°C **(B)** (bar = 50 μm), at −80°C (**C**) (bar = 20 μm) after 6 months. **(D)** Fresh pollen germination of cv. Budimka at +20°C (bar = 50 μm). **(E)** Pollen germination of cv. Budimka on −20°C and on −80°C (**F**) after 6 months (bars = 100 μm).

Fresh pollen showed a high percentage of germination and viability for all cultivars. Pollen germination rate ranged from 60% in cv. Kožara to 88% in cv. Budimka (Table 1), while pollen viability rate ranged from 59% in cv. Kožara to 98% in cv. Budimka at 0 months of storage (Table 2). Fresh pollen of cv. Budimka showed significantly higher viability and germination rates than the pollen of other cultivars (Figure 1D). Interestingly, a greater variation was observed among the cultivars in the pollen viability than in pollen germination rates (Tables 1, 2).

**Table 1:** Effect of different storage temperatures and duration on *in vitro* pollen germination of four autochthon apple cultivars.

**Cultivars**	**Temperature**	**0 month of storage**	**1 month of storage**	**2 month of storage**	**3 month of storage**	**4 month of storage**	**5 month of storage**	**6 month of storage**	**Anova *P*-Value**
Petrovača	+20°C	68 ± 3.8b/A	24 ± 2.2h/B	14 ± 1.2g/C	7 ± 0.7h/D	5 ± 0.6i/E	3 ± 0.3g/F	2 ± 0.2i/G	≤0.0001
	+4°C	68 ± 3.8b/A	55 ± 4.9c/B	40 ± 3.5c/C	26 ± 2.3d/D	21 ± 1.8f/E	2 ± 0.2h/F	6 ± 0.5f/G	≤0.0001
	−20°C	68 ± 3.8b/A	63 ± 4.0b/B	57 ± 4.6b/B	53 ± 5.1bc/C	49 ± 4.3c/C	46 ± 5.3b/C	44 ± 6.2b/C	≤0.0001
	−80°C	68 ± 3.8b/A	64 ± 3.2b/B	61 ± 4.8b/B	59 ± 3.3b/B	55 ± 3.2b/BC	51 ± 4.9c/BC	47 ± 3.3b/C	≤0.0001
Budimka	+20°C	88 ± 7.9a/A	40 ± 3.7e/B	26 ± 2.1e/C	15 ± 1.3g/D	11 ± 1.0h/E	7 ± 0.6e/F	4 ± 0.5g/G	≤0.0001
	+4°C	88 ± 7.9a/A	66 ± 6.5b/B	55 ± 5.2b/C	39 ± 3.4c/D	29 ± 2.5e/E	20 ± 1.9d/F	13 ± 1.2e/G	≤0.0001
	−20°C	88 ± 7.9a/A	69 ± 6.8b/B	67 ± 5.1a/B	66 ± 5.9a/B	64 ± 4.1a/B	59 ± 3.7a/C	56 ± 5.4a/C	≤0.0001
	−80°C	88 ± 7.9a/A	81 ± 6.7a/B	72 ± 5.7a/C	70 ± 6.4a/C	68 ± 6.5a/C	61 ± 6.0a/D	59 ± 5.5a/D	≤0.0001
Kolačara Pozna	+20°C	64 ± 3.4c/A	33 ± 3.1f/B	9 ± 0.8h/C	5 ± 0.6i/D	3 ± 0.3j/E	2 ± 0.2h/F	1 ± 0.1j/G	≤0.0001
	+4°C	64 ± 3.4c/A	46 ± 4.4d/B	30 ± 3.2d/C	21 ± 1.8e/D	14 ± 1.3g/E	5 ± 0.5f/F	3 ± 0.3h/G	≤0.0001
	−20°C	64 ± 3.4c/A	55 ± 5.2c/B	52 ± 5.0b/B	50 ± 4.5bc/BC	47 ± 4.2c/BC	45 ± 4.2b/C	36 ± 3.8c/D	≤0.0001
	−80°C	64 ± 3.4c/A	56 ± 5.4c/B	55 ± 5.1b/B	52 ± 4.8bc/B	49 ± 4.6c/BC	47 ± 4.3b/C	39 ± 4.0c/D	≤0.0001
Kožara	+20°C	60 ± 3.1d/A	28 ± 2.6g/B	6 ± 0.7i/C	3 ± 0.3j/D	2 ± 0.2k/E	1 ± 0.1i/F	1 ± 0.1j/G	≤0.0001
	+4°C	60 ± 3.1d/A	34 ± 3.2f/B	20 ± 1.8f/C	17 ± 1.5f/D	11 ± 1.0g/E	3 ± 0.3g/F	2 ± 0.2i/G	≤0.0001
	−20°C	60 ± 3.1d/A	50 ± 3.8cd/B	45 ± 4.1bc/C	42 ± 4.1c/CD	38 ± 3.7d/CD	35 ± 4.2c/D	31 ± 4.7d/D	≤0.0001
	−80°C	60 ± 314d/A	51 ± 3.6cd/B	48 ± 4.4bc/B	43 ± 4.2c/C	41 ± 4.2d/C	38 ± 3.4c/CD	34 ± 3.3d/D	≤0.0001
	Anova *P*-value	≤0.001	≤0.0001	≤0.0001	≤0.0001	≤0.0001	≤0.0001	≤0.0001	

*Means followed by the same lower case letters in a column and capital letters in a row are not significantly different by an LSD test (p ≤ 0.05)*.

**Table 2:** Effect of different storage temperatures and duration on *in vitro* pollen viability of four autochthon apple cultivars.

**Cultivars**	**Temperature**	**0 month of storage**	**1 month of storage**	**2 month of storage**	**3 month of storage**	**4 month of storage**	**5 month of storage**	**6 month of storage**	**Anova *P*-Value**
Petrovača	+20°C	79 ± 6.1b/A	34 ± 3.0f/B	22 ± 1.9f/C	18 ± 1.6g/D	15 ± 2.4h/E	11 ± 1.4k/F	9 ± 1.0i/G	≤0.0001
	+4°C	79 ± 6.1b/A	65 ± 5.4c/B	30 ± 2.7e/C	28 ± 2.4e/C	26 ± 2.3f/D	22 ± 2.5h/E	19 ± 2.1f/E	≤0.0001
	−20°C	79 ± 6.1b/A	74 ± 7.8b/A	70 ± 3.8b/B	68 ± 3.6b/B	66 ± 3.1b/B	62 ± 3.1c/C	60 ± 4.3b/C	≤0.0001
	−80°C	79 ± 6.1b/A	75 ± 6.4b/A	72 ± 5.1b/A	70 ± 4.2b/AB	69 ± 4.0b/AB	67 ± 3.0b/AB	63 ± 3.3b/B	≤0.0001
Budimka	+20°C	98 ±8.7a/A	55 ± 5.3d/B	30 ± 2.4e/C	24 ± 2.6f/D	20 ± 2.1g/E	16 ± 1.9i/F	12 ± 1.4g/G	≤0.0001
	+4°C	98 ± 8.7a/A	72 ± 7.3b/B	56 ± 3.2cd/C	48 ± 4.5d/D	35 ± 3.1e/E	30 ± 3.4g/F	28 ± 2.9e/F	≤0.0001
	−20°C	98 ± 8.7a/A	95 ± 9.7a/A	90 ± 8.9a/A	86 ± 6.8a/AB	80 ± 5.2ab/B	79 ± 6.2a/B	73 ± 7.6a/B	≤0.0001
	−80°C	98 ± 8.7a/A	96 ± 8.9a/A	93 ± 7.7a/A	90 ± 5.3a/A	86 ± 3.7a/AB	81 ± 3.5a/B	79 ± 4.1a/B	≤0.0001
Kolačara pozna	+20°C	68 ± 6.3c/A	41 ± 4.3e/B	17 ± 1.5g/C	13 ± 1.4h/D	10 ± 1.4i/E	9 ± 0.9k/F	7 ± 0.8j/G	≤0.0001
	+4°C	68 ± 6.3c/A	46 ± 4.5e/B	30 ± 2.5e/C	21 ± 2.3f/D	15 ± 1.6h/E	14 ± 1.5j/EF	13 ± 1.5g/F	≤0.0001
	−20°C	68 ± 6.3c/A	65 ± 5.6c/A	60 ± 5.0c/AB	58 ± 3.2c/AB	57 ± 5.6c/AB	56 ± 3.8d/AB	51 ± 3.4c/B	≤0.0001
	−80°C	68 ± 6.3c/A	66 ± 6.7c/A	62 ± 5.8c/A	59 ± 3.1c/AB	58 ± 3.9c/AB	57 ± 3.9d/B	56 ± 3.7c/B	≤0.0001
Kožara	+20°C	59 ± 5.6d/A	37 ± 3.9f/B	12 ± 1.3h/C	9 ± 0.8i/D	7 ± 0.9j/E	6 ± 0.7l/F	5 ± 5.1k/G	≤0.0001
	+4°C	59 ± 5.6d/A	40 ± 4.1e/B	33 ± 3.1e/C	28 ± 2.5e/D	17 ± 1.8h/E	14 ± 1.6j/F	11 ± 1.2g/G	≤0.0001
	−20°C	59 ± 5.6d/A	56 ± 5.7d/A	52 ± 4.7d/AB	49 ± 3.2d/AB	48 ± 2.7d/AB	44 ± 3.7f/B	40 ± 4.2 d/B	≤0.0001
	−80°C	59 ± 5.6d/A	58 ± 6.0d/A	57 ± 3.2cd/A	54 ± 3.1cd/A	50 ± 3.8d/B	49 ± 3.1e/B	44 ± 4.6d/C	≤0.0001
	Anova *P*-value	≤0.0001	≤0.0001	≤0.0001	≤0.0001	≤0.0001	≤0.0001	≤0.0001	

*Means followed by the same lower case letters in a column and capital letters in a row are not significantly different by the LSD test (p ≤ 0.05)*.

### Pollen Characteristics at Room Temperature

Germination and viability of pollen stored at room temperature decreased over time. So, pollen of all apple cultivars lost more than 70 and 90% of germination ability and viability after 2 and 6 months of storage at room temperature, respectively (Tables 1, 2, Figure 1A). The decrease in pollen germination and viability rates was significantly faster at room temperature than at lower temperatures. Storage at 4°C prolonged the pollen viability and germinability 1–5 fold compared with room temperature, depending on cultivars and treatment duration (Figure 1B).

### Pollen Characteristics at Low Temperature

Pollen longevity was further significantly extended when stored at −20 and −80°C (*P* ≤ 0.0001; Tables 1, 2, Figures 1C,E,F); however, pollen of all cultivars stored at −20°C showed similar germination and viability rates as pollen stored at −80°C (Figures 1E,F).

**Figure 2:**
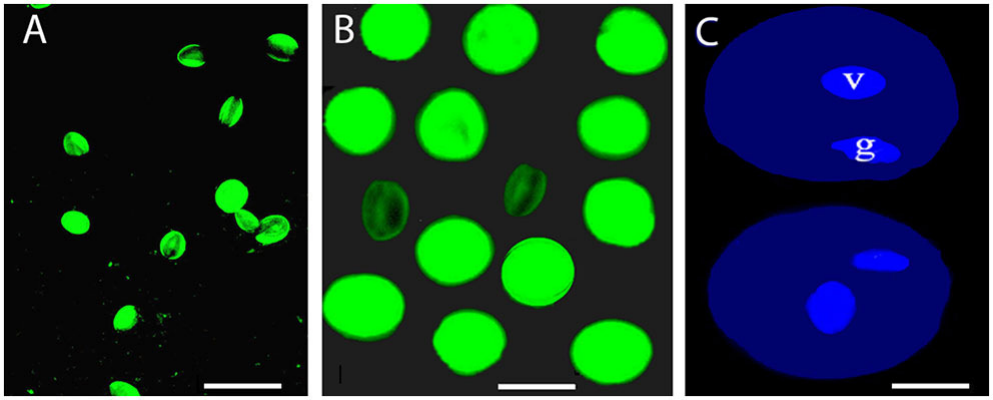
Pollen viability after storage treatment. **(A)** Pollen viability of cv. Kožara (bar = 50 μm) and cv. Budimka **(B)** after 6 months of storage at −80°C (bar = 20 μm). **(C)** Vegetative (v) and generative (g) nucleus of fresh cv. Budimka pollen after staining with DAPI (bar =10 μm).

After 6 months of storage, pollen of cv. Budimka kept about 56 and 59% of germination and 73 and 79% of viability when stored at −20 and −80°C, respectively (Tables 1, 2, Figure 2B), The germination rates were 14–15 fold higher compared with germination rates in pollen stored at room temperature for the same period (*P* ≤ 0.0001).

A significant difference between pollen stored at −20 and 4°C for all cultivars was noticed (*P* ≤ 0.0001, of each); however, no statistical differences in pollen germination rates were observed between pollen stored at −20 and −80°C for all cultivars, after 6 months of storage. In addition, pollen of all cultivars showed a 0.3–7 fold higher viability than germination at the same temperature conditions, after 6 months of storage (Tables 1, 2); however, both pollen germination and viability significantly declined throughout the treatment in all cultivars under all storage temperatures (Tables 1, 2, Figure 1, Figures 2A,B).

Data analysis showed that the effects of apple cultivar and the interactions between cultivar, storage temperature, and storage duration on pollen germination and pollen viability rates were significant (*P* < 0.001; Table 3).

**Table 3:** Mixed factorial design showing the effect of storage temperature and storage duration on pollen germination and viability of four Serbian autochthon apple cultivars.

**Source of variation**	***df***	**Pollen germination**		**Pollen viability**	
		***F*-Value**	***P***	***F*-Value**	***P***
Cultivar (A)	3	7,085	≤0.0001	7,710	≤0.0001
Storage duration (B)	6	558	≤0.0001	616	≤0.0001
Temperature (C)	3	148	≤0.001	250	≤0.001
A × B	18	104	≤0.0001	211	≤0.0001
A × C	9	244	≤0.001	519	≤0.001
B × C	18	348	≤0.001	452	≤0.001
A × B × C	54	66	≤0.001	78	≤0.001

Storage of viable pollen of Serbian autochthon apple cultivars for 6 months at −20 to −80°C is adequate for allowing breeders to efficiently carry out hybridization of germplasm flowering at different times and locations.

### Pollen Nuclear Status

An analysis of the nuclei number stained with DAPI showed that mature pollen grains of all cultivars were binucleate (Figure 2C).

## Discussion

### Viability and Germinability in Storage Pollen

Since there are no reports of temperature influence on long-term storage of pollen of Serbian apple cultivars on its viability, the present report aimed at providing information on the best storage conditions of pollen to be further used for raising the fertilization potentials of selected autochthon apple cultivars. These results showed that viability and pollen germination varied significantly depending on cultivar, storage conditions, and tests used for germination assessment. The results obtained in the present study are in agreement with results obtained by other authors (Osborne et al., 1992; Lora et al., 2006; Masum-Akond et al., 2012; Dutta et al., 2013; Novara et al., 2017; Yuan et al., 2018).

Variations in pollen longevity among plant species have been attributed to the difference in desiccation tolerance of the pollen (Song and Tachibana, 2007). Pollen of most Serbian autochthon apple cultivars lost viability (up to 12–30%) and germination potential (up to 6–26%) after 2 months at room temperature. These cultivars keep their pollen germination at 31–66% and 35–69% if stored at −20 and −80°C, respectively, for 6 months.

Pollen longevity has been reported to be extended by storing at lower temperatures (4, −20, and −80°C) in other plant species, e.g., in almond (Martínez-Gómez et al., 2002), cherimoya (Lora et al., 2006), mango (Dutta et al., 2013), hazel (Novara et al., 2017), date palm (Maryam et al., 2017), and many others.

Also, Dutta et al. (2013) and Novara et al. (2017) reported that the longevity of pollen of mango and hazel may retain viability for years with low-temperature storage, but pollen viability was generally not extended at the lowest temperature tested in their study (−20°C). This indicates that harmful physical and chemical changes proceed gradually in refrigerator-stored dry pollen (Hanna and Towill, 1995). Studies have indicated that pollen spoilage during aging involves disarranged intracellular integrity, decreased activity of enzymes, accumulation of free radicals, and de-esterification and lipid peroxidation leading to increased leakage of cellular components upon rehydration **(**Taylor and Hepler, 1997**)**. It has been shown that the aging-mediated membrane damage of pollen is not associated with protein denaturation (Wolkers and Hoekstra, 1995). Reduction in the viability of tomato pollen during long-term dry storage in a freezer involves a decline in the capacity to enhance gene expression for polyamine biosynthetic enzymes upon rehydration (Song and Tachibana, 2007).

The inclusion of 15% PEG in the liquid medium had a positive effect on pollen germination of the autochthon apple cultivars in the present study (data are not shown) and in a previous study on pollen germination of plum (Ćalić et al., 2013). PEG functions as an osmoticum, and it improves *in vitro* pollen germination frequency by preventing tube bursting (Ćalić et al., 2013).

### Nuclear Status of Pollen

These results showed that all apple cultivars had binucleate pollen grains. Binucleate pollen has weaker germination but longer survival time than trinucleate pollen. Plants that have binucleate pollen grains are pollinated *via* insects (Gottsberger, 1989). Trinucleate pollen is associated with higher metabolic activity and moisture content in comparison to binucleate pollen. Therefore, a common feature of trinucleate pollen is more sensitive to trauma by desiccation (Lora et al., 2006). Storage techniques with low temperature are more easily improved for desiccation-tolerant binucleate pollen than trinucleate pollen.

Since there is no difference in the pollen viability and germination of the Serbian autochthon apple cultivars after storage at −20 and −80°C for up to 6 months, there is no need to keep pollen at −80°C, as was also recommended for pollen of some other plant species, such as crape myrtle (Masum-Akond et al., 2012).

The significance of this study points to the necessity of preserving these old autochthon cultivars as unique genetic resources with enormous ecological, and potential economic value.

## Conclusion

From the results of this study, it can be concluded that the pollen of the four apple cultivars tested has different viability and germination capacity. Furthermore, germination of all apple cultivars tested is best maintained over 6 months when pollen is stored at −20 to −80°C.

The long period of this pollen viability makes it suitable for most breeding program applications.

## Data Availability Statement

The original contributions presented in the study are included in the article/supplementary material, further inquiries can be directed to the corresponding author/s.

## Author Contributions

DĆ and SZ-K conceived and designed the experiments. DĆ, JM, MB, RM, and SZ-K performed the experiments. JM, MB, DĆ, and SZ-K analyzed the data. DĆ and SZ-K wrote the manuscript. All authors have read the manuscript and approved it for submission.

## Conflict of Interest

The authors declare that the research was conducted in the absence of any commercial or financial relationships that could be construed as a potential conflict of interest.

## Publisher's Note

All claims expressed in this article are solely those of the authors and do not necessarily represent those of their affiliated organizations, or those of the publisher, the editors and the reviewers. Any product that may be evaluated in this article, or claim that may be made by its manufacturer, is not guaranteed or endorsed by the publisher.
